# Supplementary material for: The interactome of intact mitochondria by cross-linking mass spectrometry provides evidence for coexisting respiratory supercomplexes
Source: Mol Cell Proteomics. 2017 Dec 8;17(2):216–32. doi: 10.1074/mcp.RA117.000470 (PMC5795388; doi:10.1074/mcp.RA117.000470)
Supplement: Supplemental Data [file supp_RA117.000470_133922_0_supp_23978_fzffwf.zip › spectra_annotation/mito_spectra_annotation/144-2-7-1-9-1.pdf]

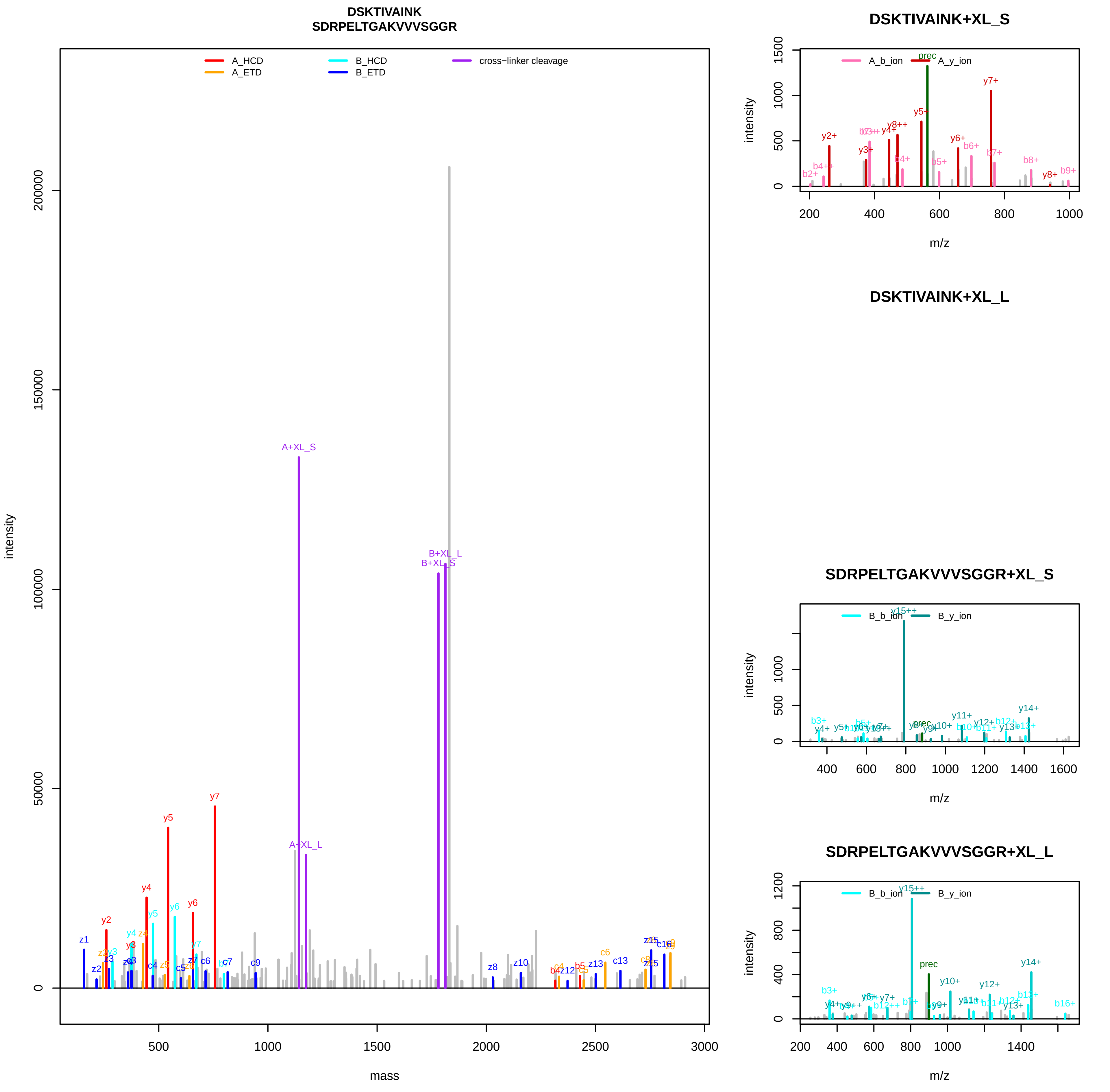
DSKTIVAINK
SDRPELTGAKVVVSGGR
A_HCD
A_ETD
B_HCD
B_ETD
cross−linker cleavage
A+XL_S
B+XL_L
B+XL_S
A+XL_L
y2
y3
y4
y5
y6
y7
z1
z2
z3
c4
c5
c6
c7
c9
z8
z10
z12
z13
c13
z15
c16
intensity
mass
0
50000
100000
150000
200000
500
1000
1500
2000
2500
3000
DSKTIVAINK+XL_S
A_b_ion
A_y_ion
prec
m/z
DSKTIVAINK+XL_L
SDRPELTGAKVVVSGGR+XL_S
B_b_ion
B_y_ion
y15++
y14+
SDRPELTGAKVVVSGGR+XL_L
